# Sodium butyrate attenuates angiotensin II‐induced cardiac hypertrophy by inhibiting COX2/PGE2 pathway via a HDAC5/HDAC6‐dependent mechanism

**DOI:** 10.1111/jcmm.14684

**Published:** 2019-09-29

**Authors:** Linlin Zhang, Mokan Deng, Aihua Lu, Yanting Chen, Yang Chen, Chunying Wu, Zhi Tan, Krishna M. Boini, Tianxin Yang, Qing Zhu, Lei Wang

**Affiliations:** ^1^ Institue of Hypertension Sun Yat‐sen University School of Medicine Guangzhou China; ^2^ School of Pharmaceutical Sciences Guangzhou University of Chinese Medicine Guangzhou China; ^3^ School of Medicine Virginia Commonwealth University Richmond VA USA; ^4^ Guangdong Metabolic Disease Research Center of Integrated Chinese and Western Medicine Guangdong Pharmaceutical University Guangzhou China

**Keywords:** Ang II, cardiac hypertrophy, cyclooxygenase‐2, prostaglandin, sodium butyrate

## Abstract

Sodium butyrate (NaBu) is reported to play important roles in a number of chronic diseases. The present work is aimed to investigate the effect of NaBu on angiotensin II (Ang II)‐induced cardiac hypertrophy and the underlying mechanism in in vivo and in vitro models. Sprague Dawley rats were infused with vehicle or Ang II (200 ng/kg/min) and orally administrated with or without NaBu (1 g/kg/d) for two weeks. Cardiac hypertrophy parameters and COX2/PGE2 pathway were analysed by real‐time PCR, ELISA, immunostaining and Western blot. The cardiomyocytes H9C2 cells were used as in vitro model to investigate the role of NaBu (2 mmol/L) in inhibition of Ang II‐induced cardiac hypertrophy. NaBu significantly attenuated Ang II‐induced increase in the mean arterial pressure. Ang II treatment remarkably increased cardiac hypertrophy as indicated by increased ratio of heart weight/body weight and enlarged cardiomyocyte size, extensive fibrosis and inflammation, as well as enhanced expression of hypertrophic markers, whereas hearts from NaBu‐treated rats exhibited a significant reduction in these hypertrophic responses. Mechanistically, NaBu inhibited the expression of COX2/PGE2 along with production of ANP and phosphorylated ERK (pERK) stimulated by Ang II in in vivo and in vitro, which was accompanied by the suppression of HDAC5 and HDAC6 activities. Additionally, knocking down the expression of HDAC5 and HDAC6 via gene‐editing strategy dramatically blocked Ang II‐induced hypertrophic responses through COX2/PGE2 pathway. These results provide solid evidence that NaBu attenuates Ang II‐induced cardiac hypertrophy by inhibiting the activation of COX2/PGE2 pathway in a HDAC5/HDAC6‐dependent manner.

## 1 INTRODUCTION

Cardiac hypertrophy, characterized by increased muscle mass accompanied by cell enlargement, is an essential compensatory response of myocytes to various pathophysiological insults including angiotensin II (Ang II).^1^, ^2^, ^3^, ^4^, ^5^ However, chronic progress to decompensated changes will eventually increase the risk of heart failure and sudden death.^6^, ^7^ Numerous mediators and signalling pathways have been found to be involved in the development of cardiac hypertrophy,^1^, ^8^, ^9^ but the underlying mechanisms contributing to the pathogenesis of cardiac hypertrophy still remain very elusive. Accumulating studies demonstrate that cyclooxygenase‐2 (COX2) signalling pathway has gained special interest as an important mediator of cardiac hypertrophy and heart diseases.^10^, ^11^, ^12^, ^13^, ^14^

Cyclooxygenase‐2 is a key enzyme catalysing the conversion of arachidonic acid to prostanoid and is found to be up‐regulated in myocardium of patients with heart failure.^15^ Accumulating evidence has demonstrated that COX2 plays an important role in modulating heart diseases.^13^, ^16^, ^17^ Inhibition of COX2 improves cardiac function after myocardial infarction in a mouse model.^11^ On the other hand, robust overexpression of COX2 in ventricular myocyte leads to mild cardiac hypertrophy with preserved cardiac function.^13^ Upon induction of COX2, neonatal ventricular myocytes preferentially produce prostaglandin E2 (PGE2).^18^ PGE2 increases total protein synthesis, promotes cell growth and elevates expression of hypertrophic marker genes such as atrial natriuretic peptide (ANP) in a dose‐dependent manner,^19^, ^20^ indicating a critical role of PGE2 in the development of cardiac hypertrophy.^14^ Selective inhibitors of COX2 decrease PGE2 production and reduce cardiac hypertrophy by decreasing myocyte cross‐sectional area and interstitial collagen fraction in the heart.^11^ The functions of PGE2 are primarily mediated by its four G protein‐coupled receptors subtypes EP1‐EP4.^21^ Among them, EP4 is the most widely distributed subtype in almost all tissues and mainly involved in PGE2‐induced protein synthesis and hypertrophy in cardiac myocytes.^14^, ^18^ Taken together, these studies suggest that COX2/PGE2 pathway may serve as a key pathogenic mechanism of cardiac hypertrophy.

Butyrate is an important bioactive metabolite produced by gut microbiota and becomes sodium butyrate (NaBu) after receiving sodium. Recent studies report that NaBu has protective effects on cardiovascular diseases including heart failure^22^, ^23^ and cardiac hypertrophy.^24^ NaBu is found to suppress the expression of hypertrophic markers,^25^ improve myocardial function and prevent cardiac remodelling in diabetic mice.^26^ NaBu is also reported to work as an inhibitor of histone deacetylases (HDACs) to modulate gene expression^27^, ^28^ and control cardiac hypertrophy in experimental rat model.^29^ Our most recent study finds that intrarenal administration of NaBu suppresses Ang II‐induced hypertension by inhibition of renal (pro)renin receptor and exerts anti‐hypertrophic effects on the heart.^30^ However, it still remains unknown whether and how systemic administration of NaBu protects against the cardiac hypertrophy induced by Ang II. Hence, the present study was designed to investigate the beneficial role of NaBu against Ang II‐induced cardiac hypertrophy and the underlying molecular mechanism.

## 2 MATERIALS AND METHODS

### 2.1 Animals

Male Sprague Dawley rats (250‐300 g; the animal centre of Beijing Vital River Laboratory Animal Technology Company) were cage‐housed and maintained in a temperature‐controlled room with 12:12 light‐dark cycle, with free access to tap water and standard rat chow. The animal protocols were approved by the Institutional Animal Care and Use Committee of Sun Yat‐sen University. The animals were killed with an excess intravenous dose of pentobarbital sodium (150 mg/kg).

### 2.2 Rat cardiac hypertrophy and administration of NaBu

Cardiac hypertrophy was induced by chronic infusion of Ang II (Sigma, 200 ng/kg/min) into rats for 2 weeks using osmotic mini‐pumps (model 2002, Alzet) implanted subcutaneously. Mean arterial blood pressure was monitored with the telemetry system (Data Science International) as we described previously. NaBu was daily administrated to rats by gavage at a dose of 1 g/kg/d. Animal groups included the following: control rats with vehicle gavage (Ctrl), Ang II‐treated rats with vehicle gavage (Ang II) and Ang II‐treated rats with NaBu gavage (Ang II‐NaBu). After 2 weeks, rats were killed and heart weight and body weight (BW) were recorded. Hearts were collected, and half of the left ventricles was fixed in 10% paraformaldehyde for section, and the rest half was frozen in liquid N_2_ and stored at −80°C.

### 2.3 Morphological and immunohistochemical analysis

The fixed cardiac tissues were paraffin‐embedded and cut into 4‐μm sections. For morphological analysis, tissue sections were stained with Periodic Acid‐Schiff or Picro‐sirius red.^31^ Bright field images of the cross‐sectional areas were taken to visualize the cell size changes in cardiomyocyte. Approximately 150 randomly selected cardiomyocytes were measured to calculate the mean cross‐sectional area using the software (Image‐Pro Plus). For collagen detection, tissue sections were stained with Picro‐sirius red and the positive staining area (red colour) was calculated using a computer program (Image‐Pro Plus). For immunostaining, slides were incubated in 3% H_2_O_2_ for 30 minutes to block endogenous peroxidase activity. The sections were incubated at room temperature for 30 minutes in PBS containing 1% BSA (Sigma) to block non‐specific binding and further incubated overnight at 4°C in a humidified chamber with antibody against ANP (Abcam) diluted 1:200 in PBS containing 1% BSA. Then, the slides were incubated for 60 minutes at room temperature in a humidified chamber with a goat anti‐rabbit IgG (H + L) HRP (Abcam) diluted 1:200 in PBS as a secondary antibody. Then, the slides were incubated with 50 μL of diaminobenzadine (BOSTER) as a substrate, counterstained with haematoxylin (Leagene), dehydrated and fixed with permount histological neutral balsam (HAORAN). The positive staining area of ANP was semiquantitatively calculated using a computer program (Image‐Pro Plus) as described previously.^32^

### 2.4 H9C2 cell line

The cardiomyocytes cell line H9C2 was purchased from the Procell Life Science & Technology Company, Wuhan, China. The H9C2 cells were grown in Dulbecco's modified Eagle Medium supplemented with 10% (V/V) foetal bovine serum (FBS) and 4500 mg/L glucose (Procell) in tissue culture flasks (Corning). The cells were pre‐treated with NaBu (2 µmol/L) for 1 hour, followed by Ang II (0.1 µmol/L, Sigma) or PGE2 (1 µmol/L, Sigma) treatment. After 24 hour, cells were harvested for gene expression analysis and the media was collected for ANP measurement.

### 2.5 Quantitative reverse transcriptase‐polymerase chain reaction

For quantitative reverse transcription‐polymerase chain reaction (qRT‐PCR), total RNA isolation was performed as previously described.^33^ Reverse transcription and SYBG qPCR were performed as the manufacturer's instructions (Roche). Primers used in this work are shown in Table 1.

**Table 1:** Sequences of the primers for qRT‐PCR

mRNA	Sense primer	Antisense primer
ANP	GAGGAGAAGATGCCGGTAG	CAGAGAGGGAGCTAAGTG
BNP	TTCCGGATCCAGGAGAGACTT	CCTAAAACAACCTCAGCCCGT
β‐MHC	CCTCGCAATATCAAGGGAAA	TACAGGTGCATCAGCTCCAG
Nlrp3	CAGAAGCTGGGGTTGGTGAA	CCCATGTCTCCAAGGGCATT
IL‐1β	TCGGCCAAGACAGGTCGCTCA	TGGTTGCCCATCAGAGGCAAGG
MCP‐1	CAGCCAGATGCAGTTAATGCC	AGCCGACTCATTGGGATCAT
COX2	CACGGACTTGCTCACTTTGT	GAACGCTTTGCGGTACTCAT
GAPDH	GTCTTCACTACCATGGAGAAGG	TCATGGATGACCTTGGCCAG
HDAC2	GCCAACCCCGCTCTGCGATC	GCCGCCTCCTTGACTGTACGC
HDAC3	CAAGACCGTGGCGTATTTCTACGA	GCCCAGTTGATGGCAATATCACAG
HDAC5	TCCCGTCCGTCTGTCTGTTA	GACATGCCATCCGACTCGTT
HDAC6	CGAGTTCTTGCAGGCACCTA	ATGCTCATAGCGGTGGATGG

### 2.6 Western blotting

Cardiac tissues and cultured H9C2 cells were lysed and subsequently sonicated in PBS containing 1% Triton X‐100, 250 µmol/L phenylmethanesulfonyl fluoride, 2 mmol/L EDTA and 5 mmol/L dithiothrietol (pH 7.5). Protein concentrations were determined by the use of coomassie reagent. A 30 µg of protein for each sample was denatured in boiling water for 10 minutes then separated by SDS‐PAGE and transferred onto PVDF membranes. The blots were blocked 1 hour with 5% non‐fat dry milk in Tris‐buffered saline, followed by overnight incubation with rabbit anti‐ANP, COX2, EP4, pERK or tERK antibodies (Abcam), Kac antibody (PTM Biolabs), HDAC5 and HDAC6 antibodies (Cell Signaling Technology) at 4°C. For internal references, the membranes were stripped and reprobed with mouse anti‐β‐actin or anti‐GAPDH antibodies. After washing with Tris‐buffered saline, membranes were incubated with goat anti‐rabbit or goat antimouse horseradish peroxidase‐conjugated secondary antibody and visualized using enhanced chemiluminescence. The intensities of blotted bands were quantified with the software Image J.

### 2.7 Enzyme immunoassay

The contents of ANP, cAMP and PGE2 in the heart tissues and H9C2 lysate or culture media were detected with commercially available enzyme immunoassay kits following the manufacturer's instructions (Gefan biotechnology). The activities of total HDACs, HDAC2, HDAC3 and HDAC6 and the active content of HDAC5 were assessed according to the manufacturer's instruction (X‐Y biotechnology).

### 2.8 Gene editing

Single‐guide RNA sequences (gRNA) for CRISPR/Cas9 gene editing of protein‐coding genes were designed by the CRISPR Design tool (http://crispr.mit.edu/). gRNA sequences were synthesized and then inserted into the BbsI‐digested plasmid pX459. HDAC5 gRNA sequences were CACCGGCCCCATGGACCCCGCGCTT (sense) and AAACAAGCGCGGGGTCCATGGGGCC (antisense). Both the sense and antisense sequences were mixed together and annealed at 95℃ 5 minutes, 75℃ 5 minutes, 55℃ 5 minutes and 25℃ 10 minutes. Then, the mixture was used to ligate into the BbsI‐digested pX459, generating a plasmid pWRHDAC5g. HDAC6 gRNA sequences were CACCGACCACGCTTCAAGGTGGCGC (sense) and AAACGCGCCACCTTGAAGCGTGGTC (antisense). The same protocol was used to get the HDAC6 editing plasmid pWRHDAC6g. gRNA sequence insertion was verified by DNA sequencing (TSINGKE).

Gene editing in H9C2 cells was carried out with Lipofectamine 3000 transfection (Invitrogen) according to manufacturer's guidelines. The transfected cells were incubated in the media with 3 µg/mL puromycin to screen out the gRNA plasmid‐containing cells. The gene‐editing cells were selected from diluted single clones, and the target protein knockout was verified by Western blot.

### 2.9 Statistics

Data are summarized as mean ± stand error. Statistics were performed using ANOVA with the Bonferroni test for multiple comparisons or by unpaired Student's *t* test for 2 comparisons. A *P*‐value below .05 was considered statistically significant.

## 3 RESULTS

### 3.1 NaBu attenuated hypertension and cardiac hypertrophy induced by Ang II

**Figure 1:**
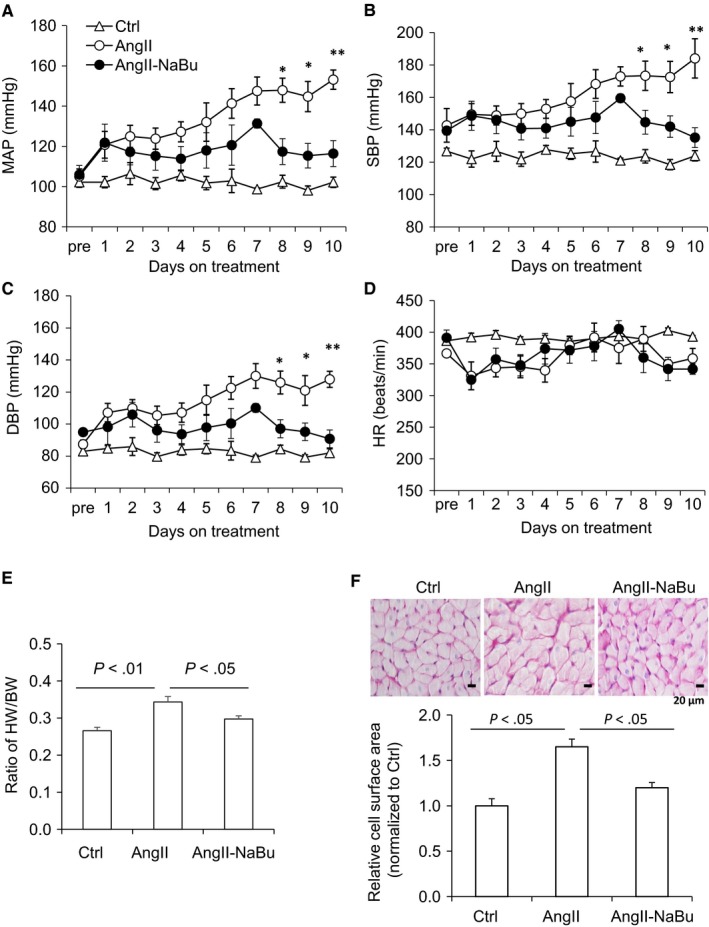
NaBu attenuated hypertension and cardiac hypertrophy induced by Ang II. SD rats were infused with vehicle or Ang II (200 ng/kg/min) and orally administrated with or without NaBu (1 g/kg/d) for 2 wk. A, Ten‐day mean arterial pressure. B, Ten‐day systolic blood pressure. C, Ten‐day diastolic blood pressure. D, Ten‐day heart rate. B, Ratio of heart weight/body weight (BW). C, Representative photomicrographs showing cross‐sectional area of cardiac myocytes stained by PAS (upper panel, scale bar: 20 µm); relative fold change of cell surface areas measurements (lower panel). * *P* < .05 or **P < .01 vs all other groups. N = 6 per group

Ang II infusion increased mean arterial blood pressure (MAP) in vehicle‐treated rats, and this increase in blood pressure was remarkably blunted in NaBu‐treated rats (Figure 1A). A similar pattern of changes was observed for systolic blood pressure (SBP) (Figure 1B) and diastolic blood pressure (DBP) (Figure 1C). In response to Ang II infusion, the heart rate (HR) in both vehicle‐treated rats and NaBu‐treated rats declined immediately compared with the control and the bradycardia persisted during the entire experimental period (Figure 1D), although on which there was no significant difference detected compared with the control, and Ang II treatment induced a greater cardiac hypertrophy in the vehicle‐treated rats as indicated by increased ratio of heart weight/body weight (BW) (Figure 1E) and enlarged sizes of cardiac myocytes (Figure 1F), which were both significantly attenuated by NaBu (Figure 1E,F).

**Figure 2:**
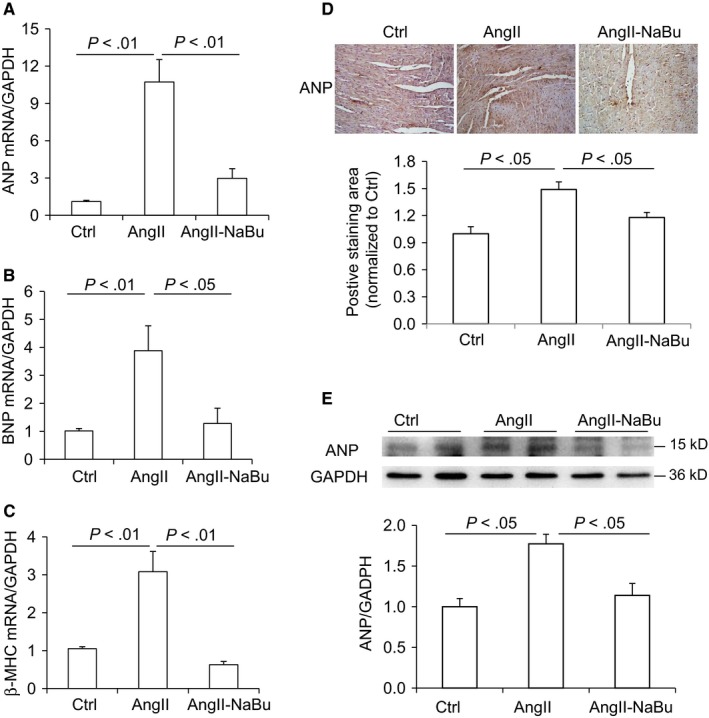
NaBu suppressed the expression of Ang II‐induced hypertrophic markers. A‐C, mRNA level of hypertrophic markers ANP, BNP and β‐MHC. D, Representative DAB IHC‐stained sections of cardiac myocyte demonstrating the expression of cardiac ANP and summarized positive staining area. E, Representative immunoblots of ANP protein levels in hearts and summarized intensities of blots. N = 6 per group

Then, the mRNA and protein levels of hypertrophic marker genes were examined in heart tissues. As shown in Figure 2A‐C, the mRNA levels of ANP, brain natriuretic peptide (BNP) and beta‐myosin heavy chain (β‐MCH) were elevated by Ang II but dramatically reduced by NaBu. Similar pattern was received on the expression levels of ANP by IHC and Western blot (Figure 2D,E). These data showed that the mRNA levels of hypertrophic marker genes were consistent with the results of ratio of HW/BW and size change in cardiac myocytes. Taken together, these results suggested that systemic administration of NaBu attenuates the cardiac hypertrophy and hypertension induced by Ang II.

### 3.2 NaBu suppressed cardiac fibrosis and inflammation induced by Ang II

**Figure 3:**
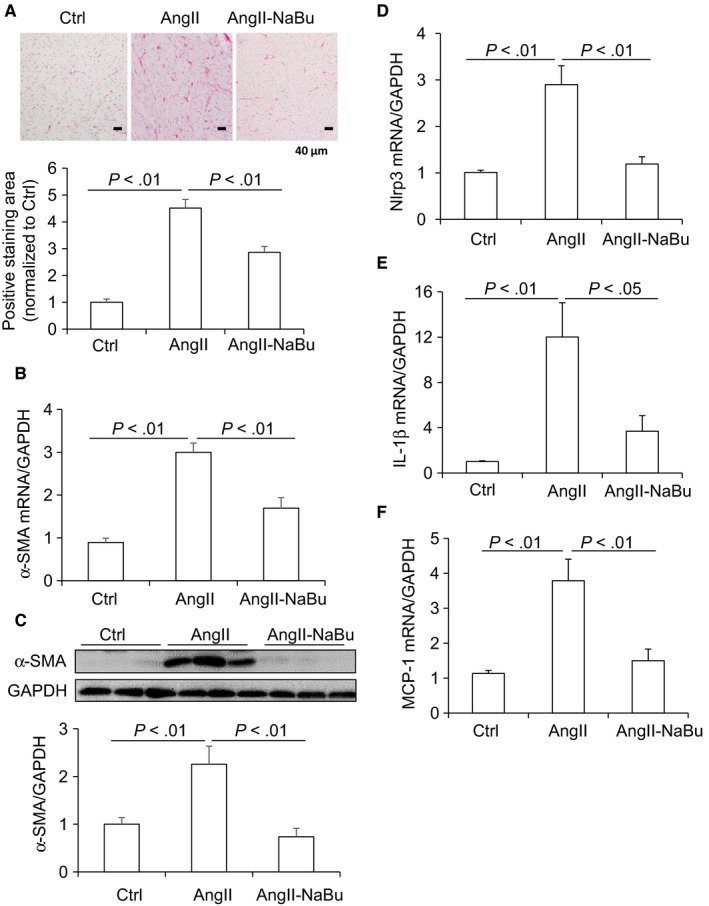
NaBu decreased Ang II‐induced cardiac fibrosis and inflammatory factors. A, representative photomicrographs showing Picro‐sirius red staining of collagens in heart (red colour) and calculated percentage of positively stained area. B, mRNA level of α‐SMA in hearts. C, Representative immunoblots of α‐SMA protein levels in hearts and summarized intensities of blots. D‐F, mRNA levels of inflammatory factors Nlrp3, IL‐1β and MCP‐1. N = 6 per group

Chronic stressors may trigger the cardiac remodelling process including fibrosis and enhanced inflammation response. Collagen I/III was detected by Picro‐sirius red staining. As shown in Figure 3A, Ang II infusion increased staining area in rat heart tissues and this Ang II‐induced increase in fibrosis was remarkably attenuated in NaBu‐treated rats. Next, the expression of α‐SMA, another index of cardiac fibrosis, was determined by qRT‐PCR and Western blot. Consistently, our results showed that Ang II increased the mRNA and protein levels of α‐SMA which were greatly attenuated by NaBu treatment (Figure 3B,C). Activation of Nlrp3 inflammasome, an intracellular danger‐sensing pathway required for the production of IL‐1β, contributes to cardiac remodelling. Ang II‐induced up‐regulation of Nlrp3 (Figure 3D) and IL‐1β (Figure 3E) was significantly inhibited by NaBu, indicating that NaBu may suppress the Nlrp3 inflammasome activation. As shown in Figure 3F, the mRNA level of monocyte chemotactic protein 1 (MCP‐1), an additional inflammatory factor induced by IL‐1β, was also found to have the similar pattern as those of Nlrp3 and IL‐1β. These results demonstrated that NaBu suppresses the cardiac fibrosis and inflammation induced by Ang II.

### 3.3 NaBu inhibited Ang II‐induced activation of COX2/PGE2 pathway along with its downstream signal cascade

**Figure 4:**
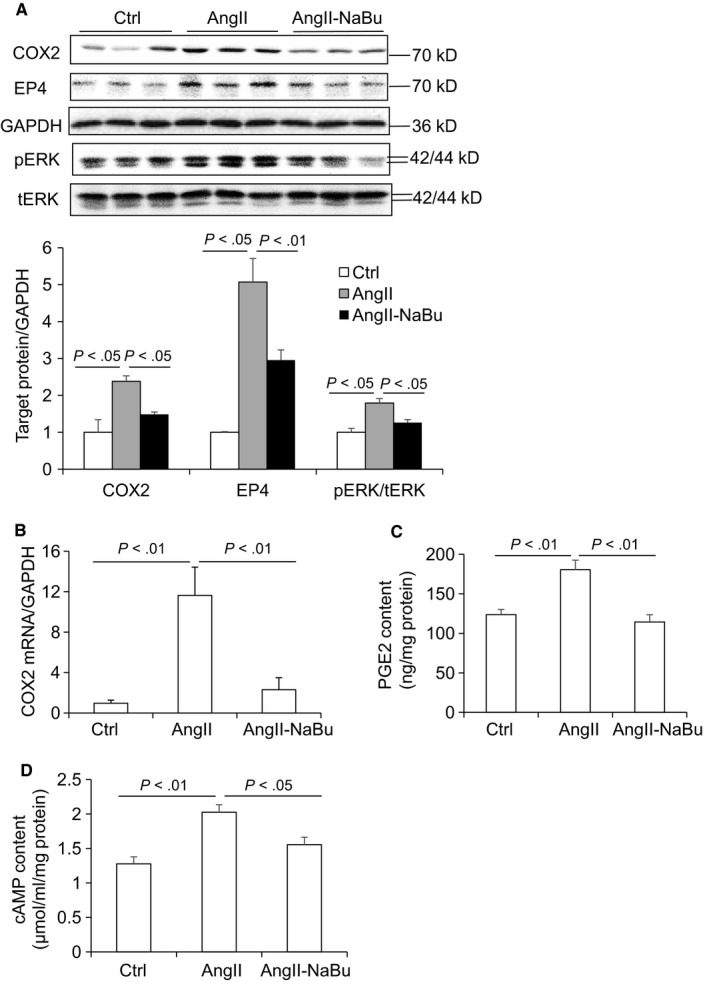
NaBu inhibited Ang II‐induced activation of COX2/PGE2 pathway along with its downstream signal cascade. A, Representative immunoblots of COX2, EP4 and pERK protein levels in hearts and summarized intensities of blots. B, mRNA level of COX2 in hearts. C, ELISA analysis of cardiac PGE2 content. D, ELISA analysis of cardiac cAMP content. N = 6 per group

It is demonstrated that COX2 and PGE2 play important roles in the regulation of cardiac hypertrophy. Stimulation of COX2/PGE2 pathway leads to the activation of downstream cascaded signal including EP4, cAMP and phosphorylation of ERK. The effect of NaBu on Ang II‐induced expression of COX2, EP4 and pERK in heart tissues was determined. Ang II infusion increased the protein and mRNA levels of COX2 which were suppressed by NaBu treatment (Figure 4A and 4). The protein expression of EP4 and pERK showed the similar pattern as that of COX2 (Figure 4A). Furthermore, PGE2 content in the heart tissues was measured by ELISA. As shown in Figure 4C, Ang II treatment led to 50% increase in PGE2 production, which was significantly inhibited by NaBu. Then, cAMP content in the heart tissues was determined by ELISA. Consistently, the cardiac cAMP content was increased by Ang II and such increase was attenuated by NaBu (Figure 4D). These results suggested that NaBu inhibits Ang II‐induced activation of COX2/PGE2 pathway along with its downstream signal cascade.

### 3.4 NaBu suppressed Ang II‐induced cardiac hypertrophy by inhibiting COX2/PGE2 pathway in H9C2 cells

**Figure 5:**
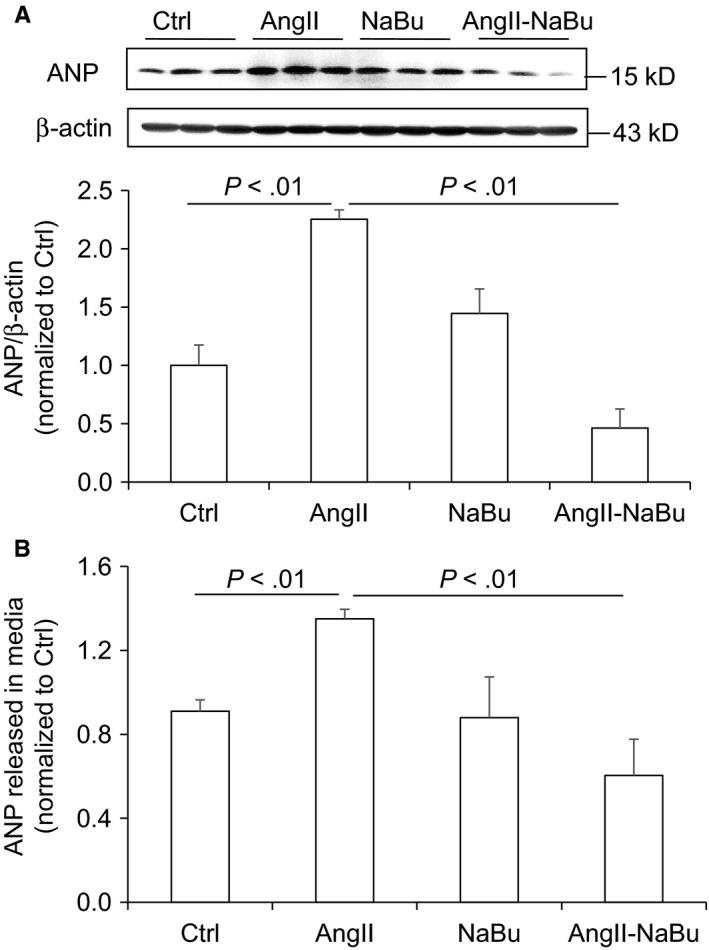
NaBu reduced Ang II‐induced ANP production in H9C2 cells. The H9C2 cells were pre‐treated with NaBu (2 µmol/L) for 1 h, followed by Ang II (0.1 µmol/L) treatment for extra 24 h A, Representative immunoblots of ANP protein levels in H9C2 cells and summarized intensities of blots. B, ELISA analysis of ANP content released in the media. N = 6 per group

**Figure 6:**
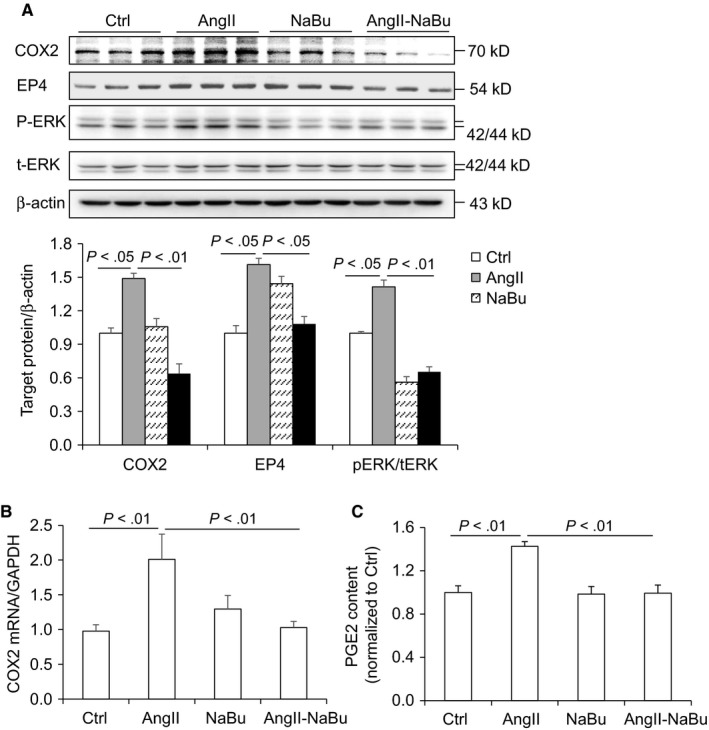
NaBu inhibited Ang II‐induced PGE2 production and expression of COX2 and pERK in H9C2 cells. The H9C2 cells were pre‐treated with NaBu (2 µmol/L) for 1 h, followed by Ang II (0.1 µmol/L) treatment for extra 24 h A, Representative immunoblots of COX2, EP4 and pERK protein levels in hearts and summarized intensities of blots. B, mRNA level of COX2. C, ELISA analysis of PGE2 content in H9C2 cells. N = 6 per group

**Figure 7:**
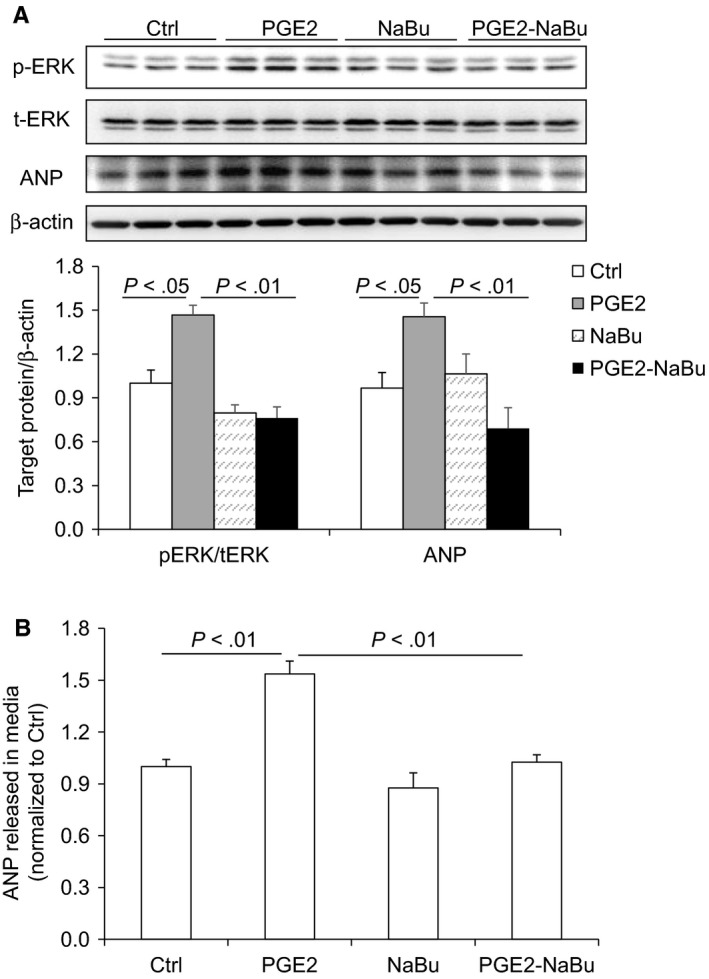
NaBu attenuated PGE2‐induced expression of pERK and ANP in H9C2 cells. The H9C2 cells were pre‐treated with NaBu (2 µmol/L) for 1 h, followed by PGE2 (1 µmol/L) treatment for extra 24 h A, Representative immunoblots of pERK and ANP protein levels and summarized intensities of blots. B, ELISA analysis of ANP content released in media. N = 6 per group

Our in vivo results suggested that NaBu might suppress cardiac hypertrophy by inhibiting the COX2/PGE2 pathway. To rule out possible effect of blood pressure change, we performed in vitro experiments to study whether NaBu inhibited Ang II‐induced hypertrophic effect in H9C2 cells. After 24‐hour treatment of 0.1 μmol/L Ang II, both the protein levels and the content of ANP released into culture media were increased and these increases were notably reduced by NaBu (Figure 5A,B). The protein and mRNA levels of COX2 in the H9C2 cells were increased by Ang II and decreased by NaBu (Figure 6A,B), which were in consistent with the above in vivo data (Figure 4A,B). Next, we determined the PGE2 content in H9C2 cells by ELISA. As shown in Figure 6C, the production of PGE2 was increased by 1.5‐fold after Ang II treatment, which was dramatically attenuated by NaBu, suggesting NaBu suppressed Ang II‐induced production of PGE2. Additionally, we determined the expression levels of PGE2 downstream molecules, EP4 and pERK. The protein levels of EP4 and pERK showed the similar pattern as that of COX2: Both were increased in Ang II‐treated cells and reduced in Ang II‐NaBu‐treated cells (Figure 6A). NaBu treatment alone slightly reduced the expression of pERK and had no effect on the production of PGE2 (Figure 6A). To confirm the role of PGE2 in mediating Ang II‐induced cardiac hypertrophy and the inhibitory effect of NaBu on cardiac hypertrophy, we treated the H9C2 cells with exogenous PGE2 in combination with NaBu. As shown in Figure 7A,B, exogenous PGE2 treatment remarkably increased the expression levels of both pERK and ANP in H9C2 cells and these increases were significantly blunted by NaBu.

Taken together, these data indicated that COX2/PGE2 pathway plays an important role in mediating cardiac hypertrophy induced by Ang II and that NaBu may suppress the cardiac hypertrophy by inhibiting COX2/PGE2 pathway along with the activation of its downstream signal cascade.

### 3.5 NaBu inhibited Ang II‐induced cardiac hypertrophy by inhibiting COX2/PGE2 pathway in a HDAC5/ HDAC6‐dependent manner

**Figure 8:**
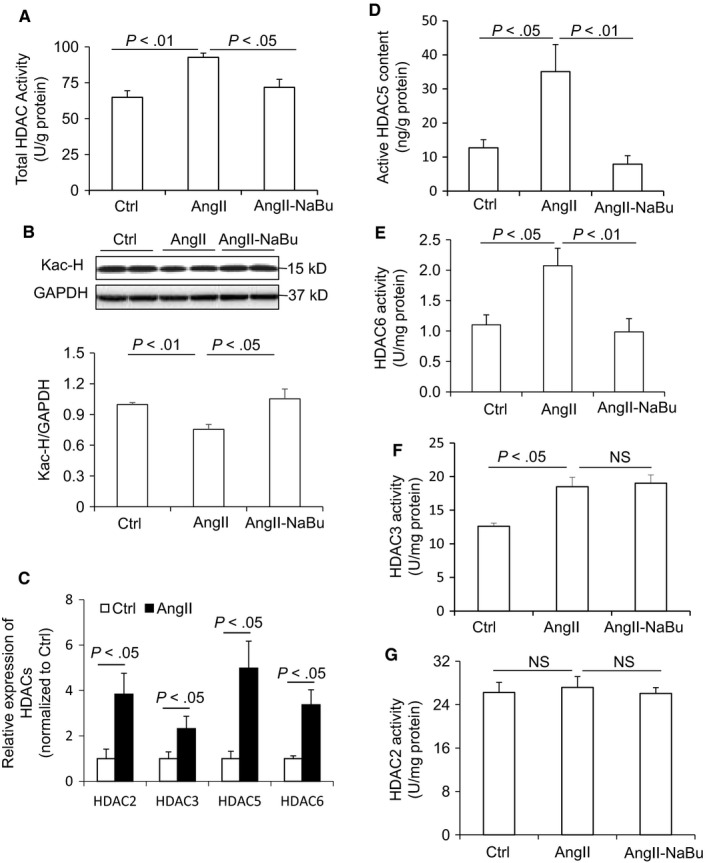
NaBu decreased the activity of HDACs in heart. A, ELISA analysis of total HDACs activity. B, Representative immunoblots of acetyl‐Histone 3 protein levels and summarized intensities of blots. C, Relative mRNA levels of HDAC2, HDAC3, HDAC5 and HDAC6. D‐G, ELISA analysis of the activity of HDAC5, HDAC6, HDAC3 and HDAC2, respectively. N = 5 per group

Since butyrate may work as an inhibitor of the class I and class II HDACs, we hypothesized that NaBu might inhibit Ang II‐induced cardiac hypertrophy through inhibition of HDACs. We first measured the total activities of HDACs by ELISA in the cardiac tissue lysates, and our results showed that the HDACs activities were much higher in Ang II‐treated rats than in Ctrl rats, indicating that HDACs were activated in Ang II‐induced cardiac hypertrophy (Figure 8A). However, the total activities of HDACs were significantly lower in Ang II‐NaBu‐treated rats compared with Ang II‐treated rats, suggesting that NaBu successfully inhibits the activities of HDACs in the cardiomyocytes (Figure 8A). To confirm the role of NaBu in histone acetylation, the protein level of acetyl‐histone 3 (Ac‐H3) was determined in heart tissues. Consistent to the result of total HDAC activity, there was a decrease in the overall histone acetylation in Ang II‐treated hearts which was significantly increased by NaBu (Figure 8B).

Then, we examined the expression levels of individual HDACs in the cardiac tissues. Our results showed that Ang II dramatically increased the mRNA levels of HDAC2, 3, 5 and 6 in rat cardiac tissues (Figure 8C), while the others (including HDAC1, 4, 7, 8, 9, 10 and 11) remained no change (data not shown). Then, the activities of HDAC2, 3, 5 and 6 were assessed by ELISA. Both the active content of HDAC5 (Figure 8D) and the activities of HDAC6 (Figure 8E) were increased by Ang II treatment and were significantly reduced in NaBu‐treated rats, indicating a functional inhibition of HDAC5 and HDAC6 by NaBu. Although the activity of HDAC3 was much higher in Ang II‐treated rats when compared with Ctrl rats, it remained no change after NaBu treatment (Figure 8F), suggesting that NaBu has no inhibitory effect on HDAC3. There was no difference in the activity of HDAC2 among these three groups (Figure 8G), indicating HDAC2 may not participate in Ang II‐induced hypertrophy.

**Figure 9:**
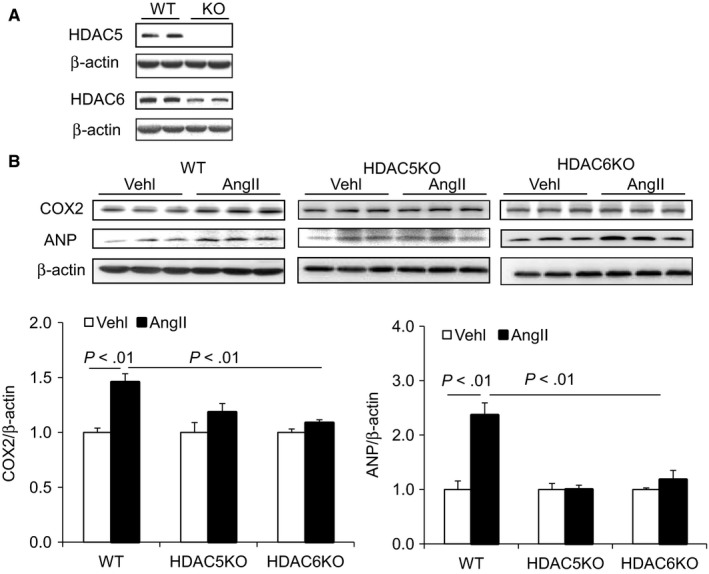
Knockdown of HDAC5 and HDAC6 suppressed Ang II‐induced expression of COX2 and ANP in H9C2 cells. A, Representative immunoblots of HDAC5 and HDAC6 protein levels from the cells of wild‐type (WT) and target genes edited.^37^ B, Representative immunoblots of COX2 and ANP protein levels in whole‐cell lysates from the WT and HDAC5 knockdown (HDAC5KO) and HDAC6 knockdown (HDAC6KO) cells subjected to Ang II treatment, and the summarized blots intensities of the indicated proteins. N = 5 per group

To further confirm the regulation of class II HDACs (HDAC5 and HDAC6) on the expression of COX2 and Ang II‐induced cardiac hypertrophy, we performed expression knockdown of HDAC5 and HDAC6 genes in H9C2 cells via *CRISPR*/*Cas9* gene‐editing plasmids. As shown in Figure 9A, the protein levels of both HDAC5 and HDAC6 were much lower in KO group than in WT group, suggesting a successful knocking down of the HDAC5 and HDAC6. Interestingly, the protein level of COX2 was significantly increased after Ang II treatment in WT cells, which was blocked in HDAC5 KO and HDAC6 KO cells (Figure 9B), indicating that inhibition of HDAC5 and HDAC6 suppresses the pathogenic response of COX2 to Ang II. The production of ANP showed the similar pattern as that of COX2 (Figure 9B), which further suggested that inhibition of HDAC5 and HDAC6 protects cardiomyocytes from Ang II‐induced cardiac hypertrophy.

Taken together, these results demonstrated that NaBu may suppress Ang II‐induced cardiac hypertrophy through COX2 signalling pathway by inhibiting the activation of HDAC5/HDAC6.

## 4 DISCUSSION

The present study investigated the protective role of NaBu on Ang II‐induced cardiac hypertrophy and further explored the underlying mechanism, with an emphasis on COX2/PGE2 pathway. In Sprague Dawley rats, oral administration of NaBu remarkably attenuated Ang II‐induced cardiac hypertrophy, cardiac fibrosis and inflammation. The anti‐hypertrophic actions of NaBu were associated with a corresponding inhibition of COX2/PGE2 pathway. We further provided in vitro evidence from cultured H9C2 cells that NaBu exerted inhibitory effects on COX2/PGE2 expression induced by Ang II, which was dependent on the deactivation of HDAC5 and HDAC6.

NaBu is generated from butyrate, an important bioactive metabolite produced by gut microbiota. We have previously reported that intrarenal administration of NaBu exerts mild anti‐hypertrophic effect on the hearts in Ang II‐induced hypertensive rats.^30^ Consistent with our results, Patel BM and colleagues have recently reported that NaBu has beneficial effect on cardiac hypertrophy in partial abdominal aortic constriction (PAAC) rat model.^29^ In the present study, we further showed that systemic administration of NaBu significantly reduced the ratio of HW/BW, diminished the sizes of cardiac myocytes, decreased the expression levels of hypertrophic marker genes and inhibited the inflammation and cardiac fibrosis (Figures 1, 2, 3), which suggested that NaBu protects against the pathogenesis of Ang II‐induced cardiac hypertrophy. These previous studies and our current results demonstrate that NaBu has a beneficial role in protecting against cardiac hypertrophy in various animal models.

It has been recognized that COX2/PGE2 pathway plays critical roles in cardiac hypertrophy.^13^, ^14^ Previous study found that overexpression of COX2 leads to substantially elevation of cardiac PGE2 and cardiomyocyte hypertrophy in a genetic mouse model.^13^ The present study showed that Ang II up‐regulated COX2 and PGE2 along with its downstream signalling molecules (EP4 and pERK) in in vivo and in vitro, which was reduced by NaBu treatment (Figures 4, 5, 6, 7). These results suggest that activation of COX2/PGE2 pathway importantly participates in the development of cardiac hypertrophy and that NaBu may contribute to the control of Ang II‐induced cardiac hypertrophy through inhibition of COX2/PGE2 pathway.

It is known that NaBu works as a non‐competitive inhibitor of HDACs,^34^, ^35^ a super family of enzymes catalysing the removal of the acetyl groups from histones. There are different classes of the HDACs, including class I HDACs (HDAC1, 2, 3 and 8), class IIa (HDAC4, 5, 7 and 9), class IIb (HDAC6 and 10), class III HDACs (Sirtuins) and class IV HDACs (HDAC11). HDACs have been well investigated in the heart for their critical roles in the regulation of pathological hypertrophy.^1^ In the current study, our data revealed that Ang II treatment increased the catalytic activities of both class I HDACs (HDAC3) and class II HDACs (HDAC5 and 6), suggesting that HDAC3, HDAC5 and HDAC6 may participate into the hypertrophic pathology in this animal model. And we also found that NaBu decreased the total HDACs activities (Figure 8A). Moreover, individual HDACs activity analysis showed that NaBu only inhibited the activities of HDAC5 and HDAC6 while had no effect on that of HDAC3 (Figure 8C‐E). These results demonstrated that deactivation of HDAC5 and HDAC6 may mediate the anti‐hypertrophic effect of NaBu in Ang II‐induced cardiac hypertrophy, which is consistent with previous study showing that HDAC6 contributes to the pathology of cardiac hypertrophy.^36^

To further confirm that NaBu may protect against Ang II‐induced cardiac hypertrophy through inhibition of HDAC5 and HDAC6, we carried out gene knockdown of HDAC5 and HDAC6 in cultured H9C2 cells via CRISPR/Cas9 strategy. As the results showed that the expression knocking down of HDAC5 and HDAC6 was accompanied by significant inhibition of COX2 and ANP expression after Ang II treatment (Figure 9). These results not only confirm the above‐mentioned importance of HDAC5/HDAC6 in Ang II‐induced cardiac hypertrophy but also provide solid in vitro evidence that inhibition of COX2/PGE2 pathway by NaBu is dependent on HDAC5 and HDAC6. In other words, the deactivation of HDAC5/HDAC6 by NaBu may serve as an upstream mediator to trigger the inhibition of COX2/PGE2 along with its downstream pathway and suppress the hypertrophic response during Ang II treatment. More importantly, this class II HDACs‐mediated inhibition of COX2/PGE2 pathway may be an possible mechanism by which NaBu fights against Ang II‐induced cardiac hypertrophy and may provide a new insight for novel therapeutic targets for cardiac hypertrophy treatment.

It should be noted that the present study does not provide an explanation for the reason why HDAC5 may have an opposing role to the other class I or II HDACs in cardiac hypertrophy.^37^ For example, cardiac HDAC2 is activated in the heart in response to pressure overload due to aortic constriction^29^; HDAC4 is increased in the heart of HFD‐fed or STZ‐induced diabetic mice^26^, ^38^; HDAC6 and 8 are up‐regulated in the heart of DOCA/salt hypertensive rat^39^; the protein levels of HDAC4, 5, 7, 6 and 10 are increased in spontaneous hypertension rats (SHRs) compared with Wistar Kyoto rats.^40^ It is interesting to note that our results about the role of HDAC5 are opposite to that of the previous study that knocking out of HDAC5 enhanced cardiac hypertrophy in a pressure over‐loaded mice model.^37^ A more recent study shows that the beneficial effect of NaBu on cardiac hypertrophy is mediated by down‐regulation of class I HDACs (specifically HDAC2) without any effect on class II HDACs such as HDAC5, in partial abdominal aortic constriction rat model^29^ based on the mRNA expression changes in HDACs after NaBu treatment. The controversy regarding the roles of HDACs may probably be due to the difference in the disease settings, stresses and analysis parameters among various experiment models. In addition, we also notice that HDAC5 is reported to be a repressor of angiogenesis in endothelial cells^41^ and NaBu is found to promote angiogenesis in the heart of HFD‐induced diabetic and myocardial infarction animals.^31^, ^38^ Angiogenesis is a cardiac remodelling response, and capillary density is observed to be reduced in pathological hypertrophy.^42^ These results indicate that HDAC5 may contribute to Ang II‐induced cardiac hypertrophy by repressing angiogenesis, which could help explain how HDAC5 functions in endothelial cells but not in cardiomyocytes. Taken together, the controversy still exists about the role of HDAC5 in cardiac hypertrophy. Thus, more detailed investigations are required regarding the role of HDAC5 during the development of cardiac hypertrophy and remodelling under different pathological settings and in different types of cells. Nevertheless, the present study provides solid in vitro evidence that deactivation of class II HDACs (HDAC5 and HDAC6) mediates the anti‐hypertrophic effect of NaBu in cardiomyocytes.

In conclusion, we demonstrated that NaBu suppresses Ang II‐induced cardiac hypertrophy through the inhibition of COX2/PGE2 pathway in a HDAC5/HDAC6‐dependent manner. Thus, the anti‐hypertrophic function of NaBu may serve as a possible therapeutic strategy to prevent Ang II‐induced cardiac hypertrophy.

## CONFLICT OF INTEREST

The authors have no conflicts of interest to declare.

## AUTHOR CONTRIBUTIONS

During the research work, Dr Lei Wang and Dr Qing Zhu designed the studies and drafted the manuscription; Linlin Zhang, Mokan Deng, Aihua Lu, Yanting Chen, Yang Chen and Chunying Wu acquired and analysed the data; Zhi Tan, Krishna M. Boini and Tianxin Yang contributed to critical manuscript review.
